# Anodic Oxidation of Tungsten under Illumination-Multi-Method Characterization and Modeling at the Molecular Level

**DOI:** 10.3390/molecules28217387

**Published:** 2023-11-01

**Authors:** Martin Bojinov, Yoanna Penkova, Iva Betova, Vasil Karastoyanov

**Affiliations:** 1 Department of Physical Chemistry, University of Chemical Technology and Metallurgy, 1756 Sofia, Bulgaria; vasko_kar@uctm.edu; 2 Institute of Electrochemistry and Energy Systems, Bulgarian Academy of Sciences, 1113 Sofia, Bulgariai.betova@iees.bas.bg (I.B.)

**Keywords:** tungsten oxide, anodic oxidation, electrochemical impedance spectroscopy, intensity modulated photocurrent spectroscopy, kinetic model

## Abstract

Tungsten oxide has received considerable attention as photo-anode in photo-assisted water splitting due to its considerable advantages such as significant light absorption in the visible region, good catalytic properties, and stability in acidic and oxidative conditions. The present paper is a first step in a detailed study of the mechanism of porous WO_3_ growth via anodic oxidation. In-situ electrochemical impedance spectroscopy (EIS) and intensity modulated photocurrent spectroscopy (IMPS) during oxidation of W illuminated with UV and visible light are employed to study the ionic and electronic processes in slightly acidic sulfate-fluoride electrolytes and a range of potentials 4–10 V. The respective responses are discussed in terms of the influence of fluoride addition on ionic and electronic process rates. A kinetic model is proposed and parameterized via regression of experimental data to the EIS and IMPS transfer functions.

## 1. Introduction

Tungsten oxide (WO_3_) has received considerable attention as photo-anode in photo-electrochemical water splitting due to its considerable advantages, such as significant light absorption in the visible region, good catalytic properties, and stability in acidic and oxidative conditions [1,2,3,4,5,6]. A low cost, relatively simple route of fabricating nanoporous tungsten oxide is based on anodization and was first described by Mukherjee et al. [7]. In addition to the promise of nanoporous WO_3_ for greatly enhancing electro- and photocatalytic properties, the electrochemical process has the advantage of being easily reproducible and under precise control. A range of papers has been dedicated to the tuning of synthesis conditions in order to maximize the light absorption and photo-catalytic properties of the obtained nanoporous layers [8,9,10,11,12,13,14,15,16]. However, all these studies have remained largely empirical, and the exact mechanism of pore nucleation and growth is still under debate.

More than a decade ago, a systematic study of the initial stages of anodic oxidation of valve metals such as W, Ti, and Mo was started in our group, aiming at the development and parameterization of a deterministic model of the process [17,18,19,20,21,22]. The proposed model was successfully validated by quantitative comparison to electrochemical, photo-electrochemical, and X-ray photoelectron spectroscopic (XPS) data and key parameter values pertinent to oxide growth, restructuring, and dissolution were estimated.

The present paper aims at characterizing the ionic and electronic processes during oxidation of W in sulfate-fluoride electrolytes under illumination using a combination of in-situ electrochemical (voltammetry and electrochemical impedance spectroscopy, EIS) and photo-electrochemical (energy spectroscopy of modulated photocurrent and intensity modulated photocurrent spectroscopy, IMPS) methods backed up with chemical information on the obtained oxides from ex-situ XPS. In that context, it is noteworthy to mention that even if IMPS was extensively used for ex-situ characterization of tungsten oxides by other authors [23,24,25,26,27,28,29,30,31], only two papers employ the method in-situ during anodization, and the response is discussed only qualitatively [32,33]. A new version of the kinetic model describing oxide growth and dissolution is parameterized by regression of experimental data to the respective EIS and IMPS transfer functions, and conclusions are drawn concerning the usefulness of the proposed approach in building a complete model of the pore initiation process.

## 2. Results

### 2.1. General Electrochemical Behavior of W in Sulfate-Fluoride Solutions

**Figure 1:**
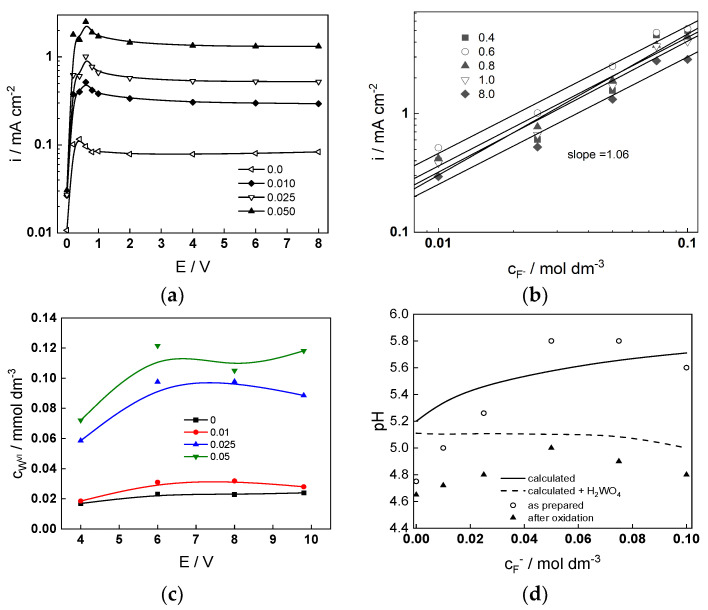
(**a**) Potentiostatic current-potential curves of W in 1 mol dm^−3^ (NH_4_)_2_SO_4_ + (0–0.05) mol dm^−3^ NH_4_F, (**b**) dependence of the steady state current density on potential and fluoride concentration, (**c**) soluble W(VI) concentration after 1 h oxidation in the range 4–10 V vs. potential and fluoride concentration (0–0.05 mol dm^−3^), (**d**) pH of the solutions before and after anodization as depending on fluoride concentration.

Potentiostatic current vs. potential curves of W in 1 mol dm^−3^ (NH_4_)_2_SO_4_ + (0–0.05) mol dm^−3^ NH_4_F are shown in Figure 1a. The regions of active dissolution (0–0.6 V), passivation (0.6–1.0 V), and passivity (1.0–8.0 V) are clearly observed. In the passivity region, the current density is quasi-independent on potential, indicating chemical dissolution of the oxide as the rate-controlling step. The current density at constant potential increases quasi-linearly with fluoride concentration (a slope of 1.06 is observed for all potentials, Figure 1b). The concentration of W(VI) in the solution after 1 h of polarization in the range of potentials 4–10 V, estimated by ICP-OES, is almost independent of potential and increases with fluoride concentration in the electrolyte, i.e., fluoride accelerates chemical dissolution of the oxide (Figure 1c). pH measurements of solutions before and after anodic oxidation indicate the formation of tungstic acid as a stable soluble product, in agreement with theoretical pH calculations (Figure 1d). It is worth mentioning that calculations with fluoro-tungstic acid were not possible due to the lack of reliable thermodynamic data.

### 2.2. Electrochemical Impedance Spectroscopy

**Figure 2:**
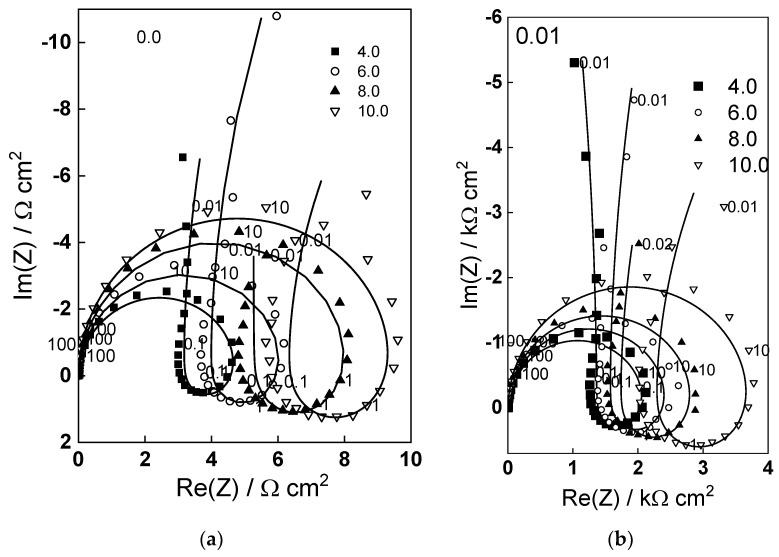
Electrochemical impedance spectra in 1 mol dm^−3^ (NH_4_)_2_SO_4_ (**a**) and 1 mol dm^−3^ (NH_4_)_2_SO_4_ +0.01 mol dm^−3^ NH_4_F (**b**) as depending on potential (illumination 365 nm). Points-experimental data, lines-best-fit calculation according to the proposed model. Parameter is frequency in Hz.

**Figure 3:**
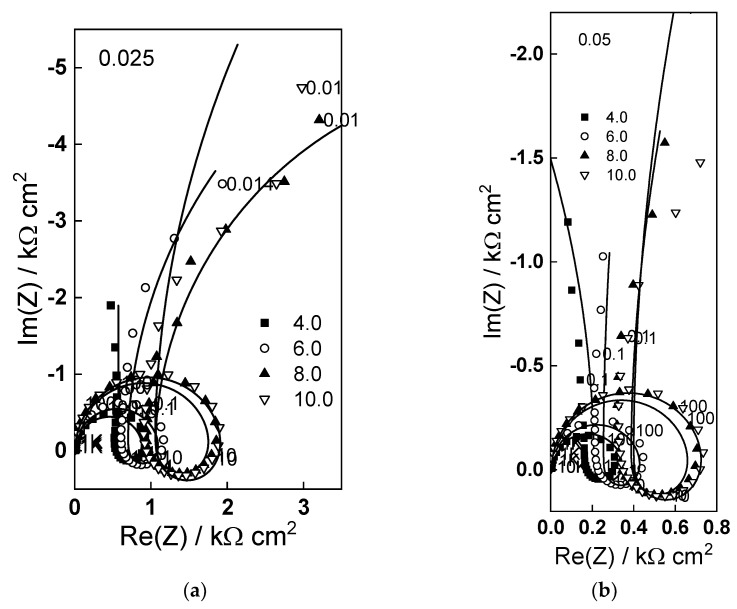
Electrochemical impedance spectra in 1 mol dm^−3^ (NH_4_)_2_SO_4_ +0.025 (**a**) and 0.05 mol dm^−3^ NH_4_F (**b**) as depending on potential (illumination 365 nm). Points-experimental data, lines-best-fit calculation according to the proposed model. Parameter is frequency in Hz.

Electrochemical impedance spectra of W oxidized in the range of potentials 4–10 V in 1 mol dm^−3^ (NH_4_)_2_SO_4_ + (0– 0.05) mol dm^−3^ NH_4_F are presented in Figure 2 and Figure 3. The spectra were measured under illumination (wavelength 365 nm). The spectra were measured typically for 3–4 h, but only the first and last measurements are shown for the sake of simplicity. It is worth mentioning that spectra measured in the absence of illumination were identical to those under illumination, i.e., no effect of light on the rate of processes during anodic oxidation is observed.

The impedance magnitude at low frequencies (e.g., 0.01 Hz) increases with potential and decreases with fluoride concentration. Three time constants are observed in the spectra, in analogy to previous measurements [17,18]. According to the qualitative interpretation proposed in those works, the high-frequency time constant is related to charge transfer and migration of current carriers in the oxide, the pseudo-inductive loop at medium frequencies corresponds to point defect interaction enhancing ionic migration, and the low-frequency time constant corresponds to the passivation reaction (its resistance is negative at potentials lower than 4 V, i.e., in the passivation region [17,18], the negative tendency being observed also in the present work with 0.05 mol dm^−3^ NH_4_F). The characteristic frequency of the time constant at intermediate frequencies increases with fluoride content, indicating that interaction of point defects of opposite sign is accelerated by fluoride.

### 2.3. Energy Spectra of the Photocurrent

**Figure 4:**
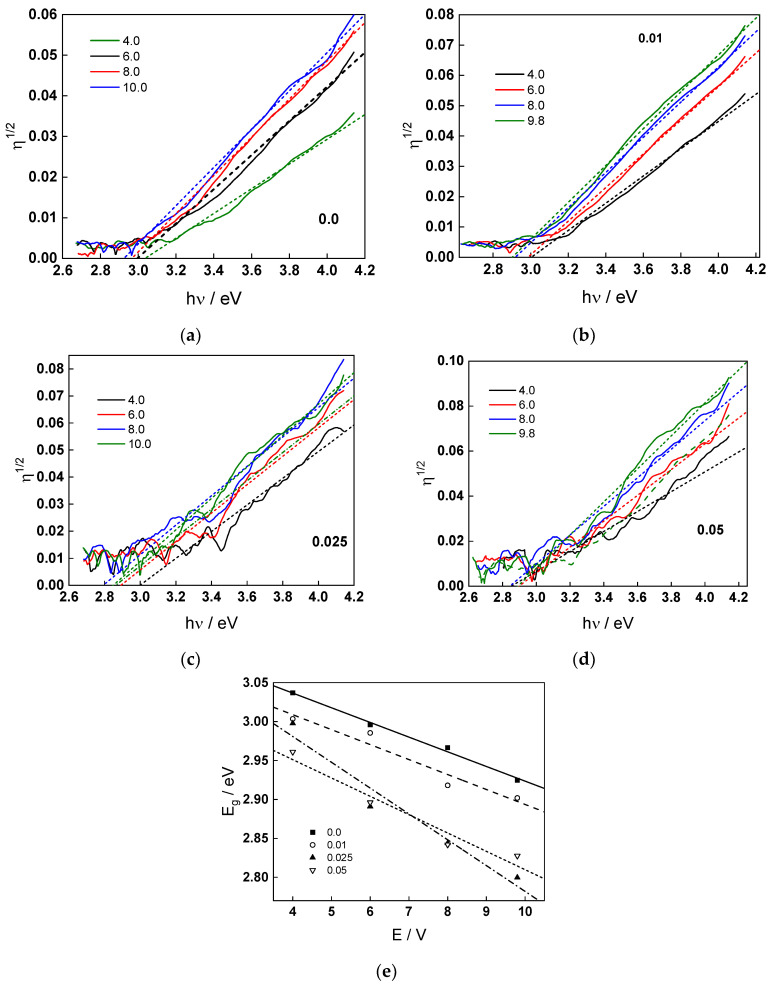
(**a**–**d**) Photocurrent spectra in coordinates allowing the estimation of indirect band gap of films formed at different potentials in 1 mol dm^−3^ (NH_4_)_2_SO_4_ + different concentrations of NH_4_F, (**e**) dependence of band gap on potential.

To further characterize the electronic nature of the oxide phase formed during anodization, energy spectra of modulated photocurrent (frequency of 12 Hz) were registered in the wavelength range 300–700 nm (Figure 4). Quantum yields were calculated from the magnitude of the photocurrent and the respective light power, and co-ordinates corresponding to indirect (phonon-assisted) transitions through the band gap were employed. Extrapolation of the η^1/2^ vs. light energy plots allowed estimating the indirect optical band gap as depending on potential and fluoride concentration. The obtained values are within the range expected for defective WO_3_ [34,35,36,37] and anodic oxides on W [11,12,13,14,15]. The apparent indirect band gap decreases with increasing measurement potential, as reported by a range of authors for compatible systems [38,39,40]. A range of explanations has been put forward for this phenomenon, such as field-induced crystallographic distortions which indirectly shift the band edges [38] and potential dependent occupancy of localized states within the bandgap [39], associated with W^5+^ and oxygen vacancies as evidenced by ex-situ analysis (see below). What is perhaps more important is that the band gap decreases with increasing fluoride content, indicating an increased defectiveness of the oxides formed in fluoride-containing solutions.

### 2.4. IMPS Response during Film Growth and Dissolution

**Figure 5:**
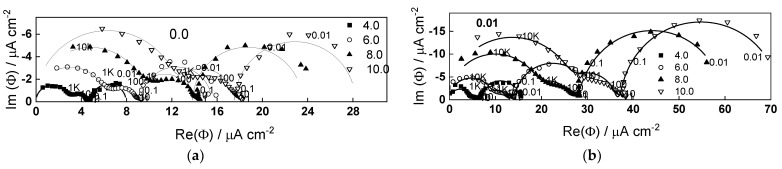
IMPS response in 1 mol dm^−3^ (NH_4_)_2_SO_4_ (**a**) and 1 mol dm^−3^ (NH_4_)_2_SO_4_ + 0.01 mol dm^−3^ NH_4_F (**b**) as depending on potential (illumination 365 nm). Points-experimental data, lines-best-fit calculation according to the proposed model. Parameter is frequency in Hz.

**Figure 6:**
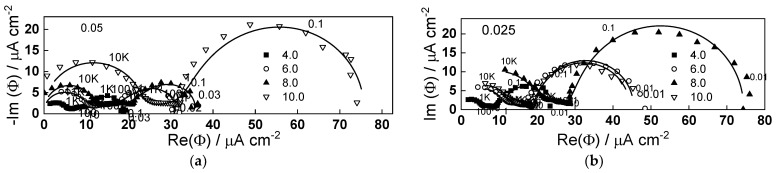
IMPS response in 1 mol dm^−3^ (NH_4_)_2_SO_4_ + 0.025 (**a**) and 0.05 mol dm^−3^ NH_4_F (**b**) as depending on potential (illumination 365 nm). Points-experimental data, lines-best-fit calculation according to the proposed model. Parameter is frequency in Hz.

To further characterize the electronic current carrier dynamics during anodic film growth on W, IMPS measurements were carried out in conditions identical to those for the EIS. The results in the range of potentials 4–10 V in 1 mol dm^−3^ (NH_4_)_2_SO_4_ + (0–0.05) mol dm^−3^ NH_4_F are shown in Figure 5 and Figure 6. The real part of the IMPS response at low frequencies increases both with potential and fluoride concentration. The spectra comprise of three time constants: the high-frequency one associated with the combination of the electrolyte resistance and the Helmholtz capacitance of the interface, and the low-frequency constants associated with charge transfer and recombination, either direct or via surface states, respectively. The relatively low characteristic frequency of the lowest frequency time constant indicates the probable participation of ionic carriers at the interface in the charge transfer process.

### 2.5. Surface Composition of the Oxides

**Figure 7:**
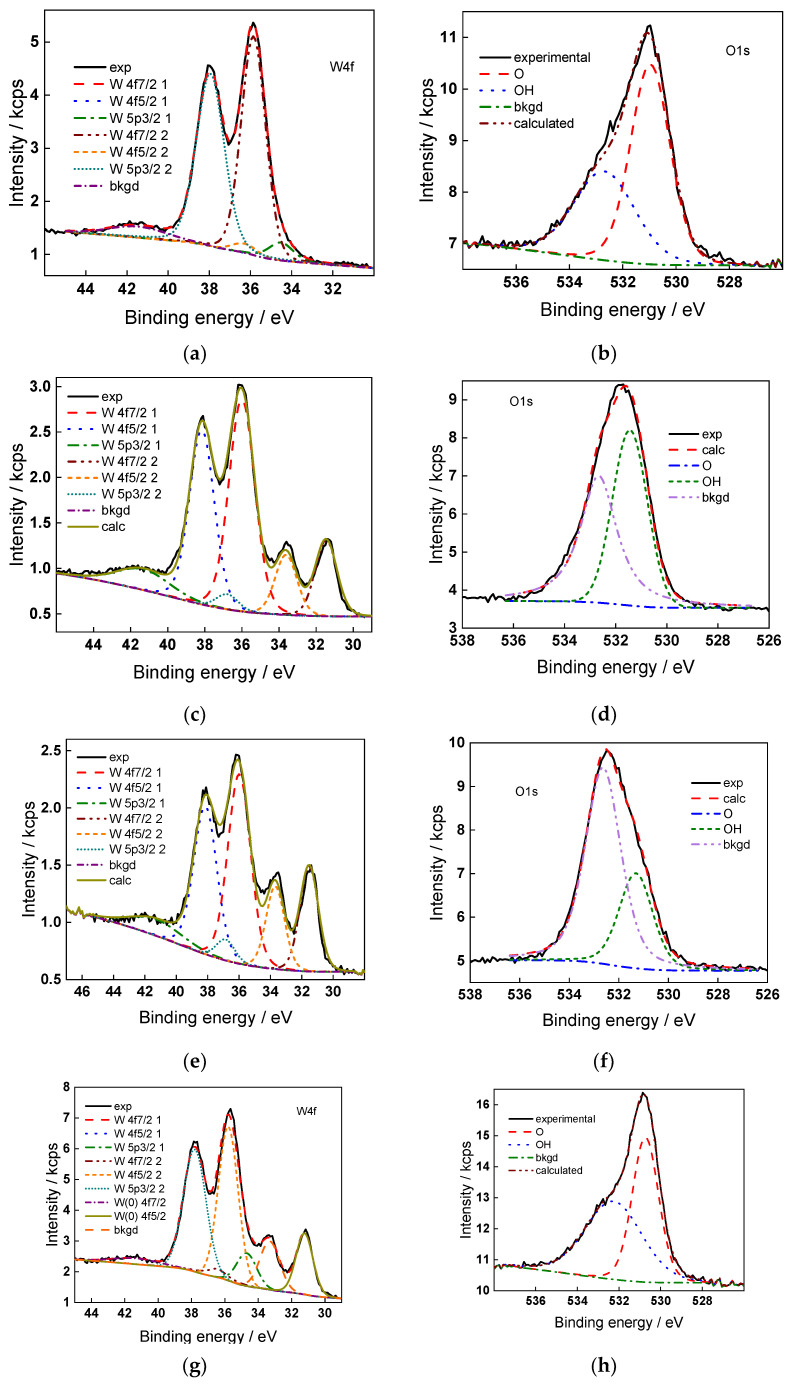
Detailed XP spectra of W4f (**a**,**c**,**e**,**g**) and O1s (**b**,**d**,**f**,**h**) for films formed in 1 mol dm^−3^ (NH_4_)_2_SO_4_ + 0 (**a**,**b**), 0.010 (**c**,**d**), 0.025 (**e**,**f**), and 0.050 (**g**,**h**) mol dm^−3^ NH_4_F at 4.0 V for 1 h.

**Figure 8:**
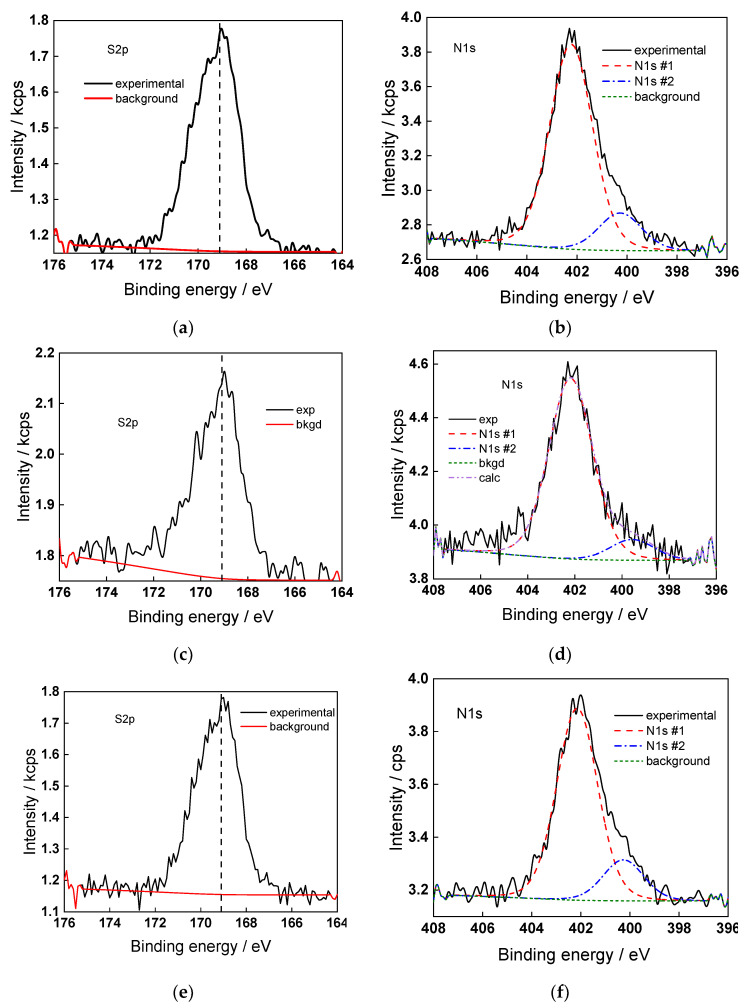
Detailed XP spectra of S2p (**a**,**c**) and N1s (**b**,**d**) for films formed in 1 mol dm^−3^ (NH_4_)_2_SO_4_ + 0.010 (**a**,**b**), 0.025 (**c**,**d**), and 0.05 (**e**,**f**) mol dm^−3^ NH_4_F at 4.0 V for 1 h. N1s #1 and #2 correspond to nitrogen as NH_4_^+^ and NH_3_, respectively.

The surface chemical composition of the oxides formed in the present conditions was characterized with XPS. Due to the fact that the detailed spectra in the interval 4–10 V are qualitatively similar, only data for a potential of 4.0 V are shown for the sake of brevity in Figure 7 (W4f and O1s spectra) and Figure 8 (N1s and S2p spectra).

W4f spectra indicate the formation of a non-stoichiometric oxide containing mainly W(VI) and some amount of W(V). Peaks of W(0) are also observed at 4.0 V, except for the films formed in sulfate, which indicates that the oxide thickness is smaller than the free path of photoelectrons through the oxide (of the order of 4 nm).

O1s spectra confirm the formation of defective WO_3_ with an O-W ratio of 2.6–2.8, with some degree of hydration at the surface. S2p spectra are consistent with a certain amount of adsorbed sulfate (ca. 8–10%), whereas N1s spectra indicate both NH_4_^+^ and NH_3_, the former predominating (ca. 14–15%). Conversely to what was found in anodic oxides on Ti formed in fluoride-containing solutions [20,21], the concentration of fluoride at the surface of anodic oxides of W was close to the detection limit.

## 3. Discussion

### 3.1. A Model for Film Growth and Tungsten Dissolution

The main assumption of the proposed model, based on the experimental results obtained is that non-stoichiometric WO_3_, containing significant amounts of W(V) in the cation sublattice is formed on W, whereas an appreciable concentration of oxygen vacancies is present in the anion sublattice. The formation of the oxide proceeds at the metal/film interface with oxidation of the metal and generation of oxygen vacancies [22]:

(1) $$W_{m}\overset{k_{O}}{\to }\left[{\mathrm{WO}}_{3}\right]\left({3V}_{O}^{••}\right){+6e}_{m}^{-}$$

The oxygen vacancies are transported through the barrier film via high-field migration and are consumed at the film/solution interface by incorporation of an oxygen ion at this interface:

(2) $$\left[{\mathrm{WO}}_{3}\right]\left({3V}_{O}^{••}\right){+3H}_{2}O\overset{k_{2O}}{\to }{\mathrm{WO}}_{3}{+6H}_{{}^{\mathrm{aq}}}^{+}$$

Dissolution of the barrier film occurs at its interface with the solution, ensuring a constant barrier film thickness at a given potential in steady state:

(3) $${\mathrm{WO}}_{3}{+6H}^{+}{+\mathrm{yF}}^{-}\overset{k_{d}}{\to }{\mathrm{WO}}_{3}F_{y}{}_{,\mathrm{aq}}^{y-}{+3H}_{2}O$$

Further, at the barrier film/solution interface, W(V) either undergoes oxidative dissolution to soluble W(VI) or transforms into a W(VI) intermediate that dissolves isovalently. Cation vacancies are generated by both reaction sequences:

(4) $$W_{w}^{V\prime }{+\mathrm{yF}}_{\mathrm{aq}}^{-}+3H_{2}O\overset{k_{2}}{\to }{\mathrm{WO}}_{3}F_{y}{}_{,\mathrm{aq}}^{y-}{+e\prime +V}_{W}{{}^{6}}^{\prime }{+6H}^{+}$$

(5) $$W_{W}^{V\prime }\overset{k_{31}}{\to }{W(\mathrm{VI})}_{\mathrm{ad}}{+e\prime +V}_{W}{{}^{6}}^{\prime }$$

(6) $${W(\mathrm{VI})}_{\mathrm{ad}}{+\mathrm{yF}}_{\mathrm{aq}}^{-}+3H_{2}O\overset{k_{32}}{\to }{\mathrm{WO}}_{3}F_{y}{}_{,\mathrm{aq}}^{y-}{+6H}^{+}$$

All the reactions at the film/solution interface leading to the formation of soluble W ions proceed with the participation of anions, whereas the reaction of formation of WO_3_ involves water as a source for oxygen. Thus, the surface is divided into dissolution and growth regions, which can lead to its local perturbation and nanopore initiation. However, it is assumed that in the present conditions, the growth/dissolution reaction is predominating, ensuring the homogeneity of the steady-state thickness at a given potential.

In addition, recombination of cation and oxygen vacancies re-creates the perfect lattice via the inverse Schottky reaction [17,18,19,20,21,22]:

(7) $${3V}_{O}^{••}{+V}_{W}{{}^{6}}^{\prime }\overset{k_{\mathrm{rec}}}{\to }\mathrm{null}$$

The material balance for cation vacancies at the barrier layer/electrolyte interface can be written in terms of the variation of the excess negative surface charge (*q_n_*) with time:

(8) $$\frac{dq_{n}}{dt}=I_{M,F/S}-I_{M}-I_{O}Sq_{n}=I_{O}S\left(\frac{I_{M,F/S}-I_{M}}{I_{O}S}-q_{n}\right),\;S=\frac{1}{F\beta_{O}}$$

*β_O_* being the surface concentration of oxygen vacancies, *I_M/_*_,*F/S*_, *I_M_* and *I_O_*-the instantaneous currents of cations at that interface, cations and anions in the bulk oxide. The charge balance for cations at the film/solution interface is expressed by the following equation:

(9) $$\frac{I_{M}}{6F}=J_{M}=k_{2}\gamma_{5}+k_{32}\gamma_{6}{}^{*}$$

*γ*_5_ and *γ*_6_* being the surface fractions occupied by W(V) and W(VI)_ad_. The material balances read as:

(10) $$\frac{\beta d\gamma_{5}}{dt}=\frac{I_{M}}{6F}-k_{2}\gamma_{5}-k_{31}\gamma_{5},\;\frac{\beta d\gamma_{6}*}{dt}=k_{31}\gamma_{5}-k_{32}\gamma_{6}*$$

*β* being the surface concentration of cation positions.

Accordingly, the steady-state current is given by the expression [22]:

(11) $$\begin{array}{l} {\bar{I}}_{}={\bar{I}}_{0}+{\bar{I}}_{M}=6F\frac{k_{32}{}^{2}{\left({\bar{k}}_{2}+{\bar{k}}_{31}\right)}^{2}+k_{d}^{2}{\left({\bar{k}}_{31}+{\bar{k}}_{32}\right)}^{2}}{\left({\bar{k}}_{31}+{\bar{k}}_{32}\right)\left[k_{32}\left({\bar{k}}_{2}+{\bar{k}}_{31}\right)+k_{d}\left({\bar{k}}_{31}+{\bar{k}}_{32}\right)\right]}\\ k_{2}=k_{2}^{0}\exp(b_{2}\bar{E}),k_{31}=k_{31}^{0}\exp(b_{31}\bar{E}),\;b_{2}=\frac{\alpha_{2}\alpha F}{RT},b_{31}=\frac{\alpha_{31}\alpha F}{RT} \end{array}$$

The steady-state value of a particular variable *x* is marked $\bar{x}$, as customary. The impedance of ion migration in the oxide is a parallel combination of the impedances due to oxygen and cation transport via vacancy mechanism. The faradic impedance due to migration of anion vacancies is derived as [22]:

(12) $$Z_{O,f}{}^{-1}=\frac{4Fa{\bar{I}}_{0}}{RT\left({\bar{L}}_{E=0}+\frac{\left(1-\alpha \right)}{\vec{E}}\bar{E}\right)}\left[(1-\alpha )+\frac{{\bar{I}}_{0}S\alpha }{j\omega +{\bar{I}}_{0}S}\right]$$

In this equation, ω is the angular frequency, *a* is the half-jump distance, α is the part of the applied potential consumed at the film/solution interface, and ${\bar{L}}_{E=0}$ is the steady-state thickness at a potential of 0 V, *F*, *R*, and *T* having their usual meanings.

In turn, the following equation is obtained for the total impedance due to anion transport:

(13) $$Z_{O}=Z_{O,f}+\frac{1}{j\omega C_{0}},C_{0}=\left({\bar{I}}_{O}+{\bar{I}}_{M}\right)\frac{\left(1-\alpha \right)}{{\bar{I}}_{O}\vec{E}}\frac{V_{m}}{4F}$$

where *C*_0_ is a pseudo-capacitance due to modulation of thickness by the ac signal, and *V_m_* is the molar volume of the oxide (taken as equal to that of WO_3_, i.e., 32.3 cm^3^ mol^−1^). The impedance of transport of cation vacancies is written in complete analogy to (12) as:

(14) $$Z_{M,f}{}^{-1}=\frac{4Fa{\bar{I}}_{M}}{RT\left({\bar{L}}_{E=0}+\frac{\left(1-\alpha \right)}{\vec{E}}\bar{E}\right)}\left[(1-\alpha )+\frac{{\bar{I}}_{0}S\alpha }{j\omega +{\bar{I}}_{0}S}\right]$$

Further, the impedance due to the dissolution of cations at the barrier layer/solution interface is derived from (9) and (10) written for a small-amplitude sine-wave perturbation around a steady state:

(15) $$\begin{array}{l} Z_{M,F/S}=\frac{1}{6Fb_{2}{\bar{k}}_{2}{\bar{\gamma }}_{5}}\left[1+\frac{b_{31}{\bar{k}}_{31}\left({\bar{k}}_{2}-{\bar{k}}_{31}\right)}{\left(1+j\left(\frac{\omega \beta {\bar{k}}_{2}b_{2}}{b_{2}k_{32}{\bar{k}}_{2}+b_{31}{\bar{k}}_{31}^{2}}\right)\right)\left(b_{2}{\bar{k}}_{2}k_{32}+b_{31}{\bar{k}}_{31}^{2}+{\bar{k}}_{31}{\bar{k}}_{2}\left(b_{2}-b_{31}\right)\right)}\right]\\ {\bar{\gamma }}_{5}=\frac{k_{32}{}^{2}\left({\bar{k}}_{2}+{\bar{k}}_{31}\right)}{\left({\bar{k}}_{31}+{\bar{k}}_{32}\right)\left[k_{32}\left({\bar{k}}_{2}+{\bar{k}}_{31}\right)+k_{d}\left({\bar{k}}_{31}+{\bar{k}}_{32}\right)\right]} \end{array}$$

The total impedance is then expressed as follows:

(16) $$Z=R_{el}+{\left[\frac{1}{Z_{e}}+\frac{1}{Z_{O}}+\frac{1}{Z_{M,f}+Z_{M,F/S}}\right]}^{-1}$$

where *R_el_* the uncompensated electrolyte resistance and *Z_e_* is the impedance of electronic properties of the oxide. This impedance was derived on the basis of steady-state profiles of predominant point defects [20] in the following form:

(17) $$Z_{e}=\frac{a}{j\omega \varepsilon \varepsilon_{0}}\ln\frac{1+j\omega \frac{RTk_{2O}}{F^{2}D_{e}k_{O}}\varepsilon \varepsilon_{0}\exp\left[\frac{{\bar{L}}_{E=0}+\frac{\left(1-\alpha \right)}{\vec{E}}\bar{E}}{a}\right]}{1+j\omega \frac{RTk_{2O}}{F^{2}D_{e}k_{O}}\varepsilon \varepsilon_{0}}$$

In the last equation, *D_e_* is the diffusion coefficient of electronic current carriers, ε is the dielectric constant of the oxide, and ε_0_ is the dielectric permittivity of free space.

**Figure 9:**
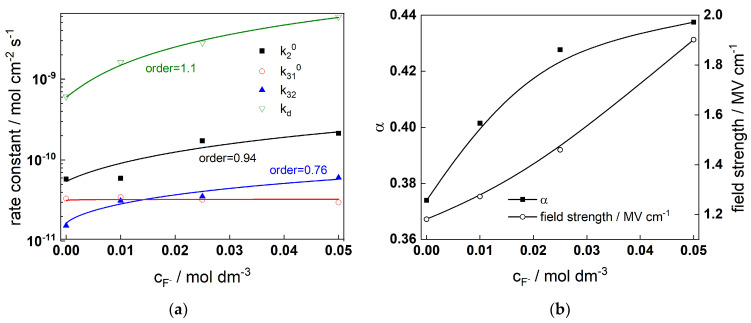
(**a**) rate constants and (**b**) field strength and polarizability of the film/solution interface estimated from regression of experimental data with respect to the model equations as depending on fluoride concentration.

Fits to Equations (12)–(17) are shown in Figure 2, Figure 3, Figure 4 and Figure 5 with solid lines and illustrate the ability of the model to account for the magnitude and the frequency distribution of the impedance spectra in all studied conditions. The main kinetic parameters are presented in Figure 9a,b as a function of fluoride concentration, whereas the parameters not influenced by fluoride are collected in Table 1. The reaction orders of the individual steps are marked in the figure and indicate the important influence of fluoride on the process rates in agreement with experiment. The increase of the field strength in the oxide with fluoride concentration is in line with the larger oxide growth rates due to higher layer dissolution rates in the presence of fluoride. Further, the importance of the part of the potential consumed as drop at the film/solution interface (α) is higher in the presence of fluoride, which once more indicates the intensification of dissolution processes at that interface.

### 3.2. Interpretation of the IMPS Response

The IMPS results are interpreted with a model featuring trapping of photo-induced holes by a surface species A to form an intermediate A^+^ (q_1_). This intermediate transforms into product A^2+^ either by capturing another hole (q_2_), or a reaction of two mobile A^+^ (q_4_) [41]. The intermediate can also absorb an electron to re-create A (q_3_):

(18) $$\begin{array}{l} h\nu \to h^{•}+e^{\prime }\\ A+h^{•}\overset{q_{1}}{\to }A^{+}\\ A^{+}+h^{•}\overset{q_{2}}{\to }A^{2+}\\ A^{+}+e^{\prime }\overset{q_{3}}{\to }A\\ A^{+}+A^{+}\overset{q_{4}}{\to }B^{2+} \end{array}$$

The transfer function has the following form:

(19) $$\begin{array}{l} \Phi (\omega )=\frac{g(k_{tr}+j\omega (C/C_{SC}))}{(k_{tr}+k_{r}+j\omega )(1+j\omega \tau_{ss})+\left[C_{ss}/(C_{SC}+C_{H})\right]\left[j\omega /(1+j\omega \tau_{ss})\right]\left[1+R_{el}C_{H}(k_{tr}+j\omega )\right]}\\ k_{tr}=\frac{q_{1}\left(\sqrt{q_{3}^{2}+8gq_{4}}-q_{3}\right)}{q_{1}+\sqrt{q_{3}^{2}+8gq_{4}}},\;k_{r}=\frac{q_{1}q_{3}}{q_{1}+\sqrt{q_{3}^{2}+8gq_{4}}} \end{array}$$

where *C_sc_* is the depletion layer capacitance (assumed to be equal to the capacitance of the oxide), *C_ss_*—surface state capacitance, *C_H_*—Helmholtz capacitance, *g*—minority carrier flux:

(20) $$g=\Phi_{0}\left[1-\frac{e^{-\alpha_{\mathrm{opt}}W}}{1+\alpha_{\mathrm{opt}}L_{p}}\right]$$

Φ_0_—incident photon flux, α_opt_—absorption coefficient of the oxide at a given wavelength of light (2 × 10^5^ cm^−1^ at 365 nm [34]), W—depletion layer width (assumed to be equal to film thickness), L_p_—hole diffusion length (ca. 150 nm [38]), and τ_ss_—time constant for charging of surface states.

**Figure 10:**
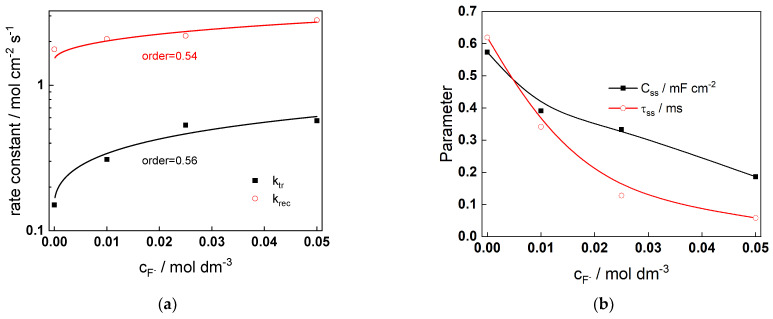
(**a**) rate constants of charge transfer and recombination and (**b**) surface state capacitance and time constant for charging the surface states estimated from regression of experimental data with respect to the model equations as depending on fluoride concentration.

The parameters estimated from regression of the experimental data to the transfer function (rate constants of charge transfer and recombination, surface state capacitance, as well as time constant for charging of surface states) are collected in Figure 10.

The influence of fluoride concentration on charge transfer and recombination reactions is also significant (reaction orders ca. 0.5), i.e., these reactions are accelerated by fluoride ions leading to an increase of the magnitude of the IMPS response in fluoride-containing solutions, as observed experimentally. While not all the kinetic parameters (q_1_, q_3_, and q_4_) can be unequivocally determined from the present data, it can be speculated from the modeling results that the following sequence of reactions takes place:

(21) $$\begin{array}{l} h\nu \to h^{•}+e^{\prime }\\ W_{W}^{V}{}^{\prime }+h^{•}\overset{q_{1}}{\to }W_{W}^{VI}\\ W_{W}^{\mathrm{VI}}+W_{W}^{\mathrm{VI}}\overset{q_{4}}{\to }2W_{\mathrm{aq}}^{6+}\\ W_{W}^{\mathrm{VI}}+e^{\prime }\overset{q_{3}}{\to }W_{W}^{V}{}^{\prime } \end{array}$$

The presence of an adsorbed intermediate acting as a surface state is confirmed by the values of the *C_ss_*, which are several times higher than those of the Helmholtz capacitance *C_H_* (the latter preserves values of 25–30 µF cm^−2^ regardless of the conditions).

In general, it can be concluded that IMPS measurements represent an important contribution to the elucidation of the mechanism of anodic oxidation of tungsten and merit wider utilization in the future.

## 4. Materials and Methods

Anodic oxidation was performed in electrolytes containing 1 mol dm^−3^ (NH_4_)_2_SO_4_ + 0, 0.1, 0.25, or 0.5 mol dm^−3^ NH_4_F at 22 ± 1 °C in naturally aerated conditions. The electrolytes were prepared from p.a. chemicals and type 2 de-ionized water. Working electrodes were cut from W sheets (99.99%, Goodfellow, Cambdrige, UK). Their pretreatment consisted of degreasing, chemical polishing for 60 s in 0.1 mol dm^−3^ KOH, and drying. A PTFE cell featuring a fused silica optical window (Edmund Optics, York, UK, transmittance 200–1000 nm) was employed for all the measurements. The 3-electrode cell featured a Pt sheet (99.9%, Goodfellow, UK) counter electrode and 3M KCl/AgCl/Ag reference electrode (LL-type, Metrohm, Herisau, Switzerland, E = 0.2 V vs. standard hydrogen electrode).

Electrochemical measurements (linear sweep voltammetry, chronoamperometry at constant potential, and EIS) were performed using a CompactStat 10800 (Ivium Technologies, Eindhoven, The Netherlands) equipped with a frequency response analysis module. Impedance spectra were measured in potentiostatic mode after a steady state current was reached, typically in a frequency range 5 mHz–22 kHz at an ac amplitude of 15 mV (rms). Linearity was verified by measuring spectra at selected potentials with amplitudes 5–20 mV, whereas causality was ensured by a Kramers–Kronig test embedded in the measurement software. Points that did not pass the test (usually at both frequency ends) were rejected.

Energy spectra of modulated photocurrent were registered by a 100 W Xe lamp (Newport 66909, Crewe, UK), a grating monochromator (Newport 77250, UK) and a 418 chopper (Bentham, Lancaster, UK) as a modulated light source (typically at 12 Hz) in the interval of light wavelengths 300–700 nm. Photocurrents were detected by a 5210EC lock-in analyzer (Signal Recovery, Oak Ridge, TN, USA) controlled by in-house designed routine on a LabView platform and coupled to a PAR 263A potentiostat (Ametek, Berwyn, PA, USA) driven by Power Suite 2.40 software. The intensity of incident light was measured with a power meter (Gamma Scientific, San Diego, CA, USA) to transform photocurrent into intensity-to-photocurrent conversion efficiency.

Intensity modulated photocurrent spectroscopy (IMPS) was performed using a CompactStat 10800 coupled to a Modulight photo-diode array source (Ivium Technologies, Eindhoven, The Netherlands) with peak wavelengths 365 and 395 nm at an incident light power of 17 mW cm^−2^, driven by IviumSoft 4.98 software. The ac amplitude of the light intensity was set at 10% of the dc value at the corresponding wavelength. Frequency range was from 5 mHz to 22 kHz. Non-linear least squares fitting of EIS and IMPS data to selected transfer functions by Levenberg–Marquardt algorithm was performed using in-house elaborated routine on an Originlab platform.

Chemical composition of the oxide films at the surface was assessed by X-ray photoelectron spectroscopy (XPS) using an Escalab II spectrometer with a monochromatic Al Kα radiation (1486.6 eV) at a base pressure of 10^−7^ Pa. Detailed spectra of C1s, N1s, O1s, F1s, S2p, and W4f were obtained at a band pass energy of 20 eV, and binding energies were calibrated vs. the C1s peak at 285.0 eV. To estimate chemical composition, spectra were fitted to Gaussian–Lorentzian peak functions after Shirley background subtraction using XPSPeak 4.1 software. The concentration of soluble tungsten generated during oxidation was measured with an ICP-OES apparatus (Prodigy, Teledyne Leeman Labs, Mason, OH, USA).

## 5. Conclusions

In the present paper, the multi-method characterization and deterministic modeling of the anodic oxidation of tungsten in sulfate-fluoride electrolytes are described. The following conclusions concerning the correspondence between experimental characterization and modeling can be drawn:

- Electrochemical data (current vs. potential curves and impedance spectra in a wide range of potentials and fluoride concentrations) give the possibility to estimate the rate constants at the oxide/electrolyte interface and the transport parameters through the oxide layer. Reaction orders vs. fluoride indicate the active participation of the anion in the oxidation and dissolution processes.
- The validity of the obtained estimates is in accordance with surface composition data obtained by XPS. Conversely to what is observed with titanium, the concentration of fluoride at the surface is rather low, i.e., it can be regarded as a catalytic species.
- Electronic carrier dynamics during film growth and dissolution is assessed using IMPS measurements in a wide range of potentials. A qualitative relationship between phenomenological parameters obtained from a generalized interpretation of the IMPS response and the sequence of charge transfer reactions at the interface is tentatively proposed.
- Further extension of this type of measurements is needed in order to quantify the relation between photo-induced charge transfer/recombination of electronic carriers, on one hand, and generation, transport, and consumption of ionic point defects, on the other.

## Figures and Tables

**Table 1:** A summary of model parameters independent of fluoride concentration.

Parameter	Value
α_2_	0.33 ± 0.011
α_31_	0.33 ± 0.010
*a*/nm	0.32 ± 0.012
*β_O_*/nmol cm^−2^	0.11 ± 0.003
*β*/nmol cm^−2^	0.3 ± 0.01

## Data Availability

The data presented in this study are available on request from the corresponding author.
